# Fabrication of Self-Oscillating Gels by Polymer Crosslinking Method and Analysis on Their Autonomous Swelling-Deswelling Behaviors

**DOI:** 10.3390/gels8050267

**Published:** 2022-04-24

**Authors:** Komi Sato, Takafumi Enomoto, Aya M. Akimoto, Ryo Yoshida

**Affiliations:** Department of Materials Engineering, School of Engineering, The University of Tokyo, 7-3-1 Hongo, Bunkyo-ku, Tokyo 113-8656, Japan; 310komi@gmail.com (K.S.); enomoto@cross.t.u-tokyo.ac.jp (T.E.); akimoto@cross.t.u-tokyo.ac.jp (A.M.A.)

**Keywords:** self-oscillating gel, polymer crosslinking method, autonomous volume oscillation

## Abstract

We have developed a new methodology for fabricating self-oscillating gels by a post-polymerization crosslinking. The method enables us to make the self-oscillating gels easily just by mixing two kinds of polymer solutions at room temperature with fast gelation. Moreover, the polymer crosslinking method has the advantage that the self-oscillating gels could be fabricated from well-defined linear polymers. We revealed that the dynamic swelling-deswelling behavior of the gels was simply affected by the net amount of the catalyst for the Belousov–Zhabotinsky reaction in the whole gels, although the equilibrium swelling behavior was influenced by the properties of the constituent linear polymers. Our results offer the opportunity to access the origin of the dynamic and equilibrium behavior of materials by the hierarchical assembly as well as enable easy microfabrication of the self-oscillating gel.

## 1. Introduction

The dynamic behavior of living organisms emerges from hierarchical assemblies of small molecules into molecular machinery, up to macroscopic scales. A molecular event, such as a simple chemical reaction, is dramatically enhanced to eventually macroscopic events through the hierarchically organized chemical networks. For example, the contraction of a muscle is induced by the hydrolysis of adenosine triphosphate bound to myosin and subsequent periodic conformational changes of myosin on the actin filament [1,2]. One of the ultimate goals for material science is the creation of artificial functional materials which realize life-like dynamic behavior driven by molecular based chemical systems. An extensive effort has been made to build the model systems of the molecular based actuators [3,4,5].

In this context, we have developed self-oscillating gels, which convert the chemical energy of the Belousov–Zhabotinsky (BZ) reaction into the periodic volumetric changes of the gels [6,7,8]. The BZ reaction is a well-known oscillatory reaction exhibiting periodic redox changes of a catalyst molecule by consuming an organic substrate, malonic acid. The self-oscillating gels are composed of poly(*N*-isopropylacrylamide) (PNIPAAm) as a thermoresponsive polymer and copolymerized ruthenium tris(2,2′-bipyridine) complex (Ru(bpy)_3_) as a catalyst for the BZ reaction. In a catalyst-free BZ solution, the self-oscillating gel induces the BZ reaction inside the gel and converts the periodic redox changes of the Ru(bpy)_3_ moiety to the macroscopic swelling-deswelling oscillation of the gel. To date, we have developed several fabrication methods of the self-oscillating gels, such as a radical copolymerization of NIPAAm and Ru(bpy)_3_ monomer [6], post-polymerization crosslinking of the self-oscillating microgels [9] and post-polymerization modification of PNIPAAm gels by Ru(bpy)_3_ derivative [10,11].

**Figure 1:**
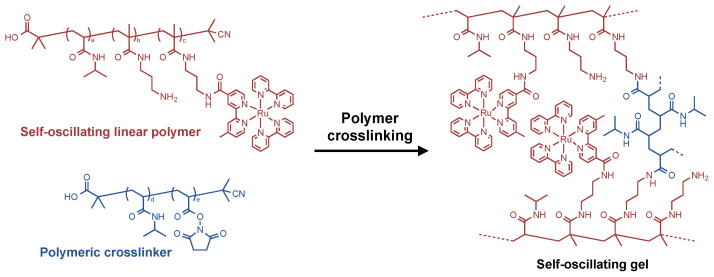
Conceptual scheme of polymer crosslinking method for fabricating self-oscillating gels.

Herein, we report a new fabrication method of the self-oscillating gels by a post-polymerization crosslinking of linear polymers. The polymer crosslinking method is realized by utilizing a condensation reaction between amino groups and *N*-hydroxysuccinimidyl (NHS) esters. We designed two linear polymers for the fabrication of the self-oscillating gels by the polymer crosslinking method, that is, a self-oscillating linear polymer and polymeric crosslinker (Figure 1). The self-oscillating linear polymer is composed of PNIPAAm with Ru(bpy)_3_ moiety and amino groups, and the polymeric crosslinker consists of PNIPAAm with NHS esters. By mixing the two solutions of the self-oscillating linear polymer and the polymeric crosslinker, in-situ crosslinking proceeds between these polymers, and the self-oscillating gels were fabricated. The polymer crosslinking method has the advantage that the self-oscillating gels could be fabricated from well-defined linear polymers. Further, this method may enable easy microfabrication of the self-oscillating gel. Thus, we could access the hierarchical effects on the self-oscillating behavior of the gels through the polymer crosslinking method. In this work, the in-situ crosslinking process was investigated by modulating the molecular weight of the polymeric crosslinkers, the feeding ratio of the crosslinking moieties and the concentration of the pre-gel polymer solution. Both equilibrium and dynamic swelling-deswelling behavior of the prepared gels were demonstrated, and the effects of the constituent polymers were discussed.

## 2. Results and Discussion

### 2.1. Preparation of Self-Oscillating Polymers and Polymer Crosslinkers

**Figure 2:**
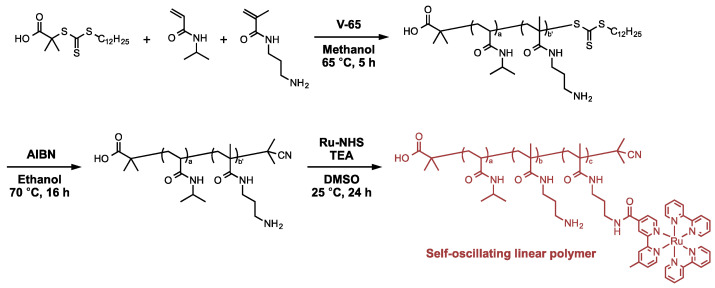
Synthetic scheme of the self-oscillating polymers.

To fabricate the self-oscillating gels by the polymer crosslinking method, we synthesized the self-oscillating linear polymers containing amino groups for the post-polymerization crosslinking (Figure 2). First, P(NIPAAm-*co*-NAPMAm) was prepared as a base copolymer via reversible addition-fragmentation chain transfer (RAFT) polymerization. Then, Ru(bpy)_3_ catalyst was conjugated to P(NIPAAm-*co*-NAPMAm) through a coupling reaction of the primary amine groups of NAPMAm with a Ru(bpy)_3_ derivative containing NHS esters (Ru-NHS). The loading amount of Ru(bpy)_3_ on P(NIPAAm-*co*-NAPMAm) was controlled by changing the feeding ratio of Ru-NHS to amino groups. We successfully synthesized three self-oscillating linear polymers (P(NIPAAm-*co*-NAPMAm-*co*-NAPMAmRu)) with the different conjugation ratios of Ru-NHS to amino groups; 10% (SOP-10), 20% (SOP-20) and 30% (SOP-30). The compositions, molecular weights and polydispersity indexes of the prepared self-oscillating linear polymers are summarized in Table 1.

**Figure 3:**
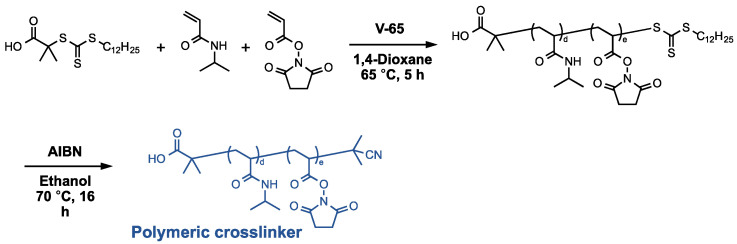
Synthetic scheme of the polymeric crosslinkers.

The polymeric crosslinkers were synthesized by RAFT polymerization of NIPAAm and *N*-acryloxysuccinimide (NAS) as monomers (Figure 3). We modulate the polymerization time and the feeding ratio of the monomers and the chain transfer agent to prepare the polymer crosslinkers with various molecular weights. The polymer crosslinkers with the different molecular weights; 10 kDa (PCL-10k), 20 kDa (PCL-20k), 40 kDa (PCL-40k) and 65 kDa (PCL-65k); were successfully synthesized. The compositions, molecular weights and polydispersity indexes of the polymer crosslinkers are summarized in Table 2.

### 2.2. LCST Analysis of Self-Oscillating Polymers

**Figure 4:**
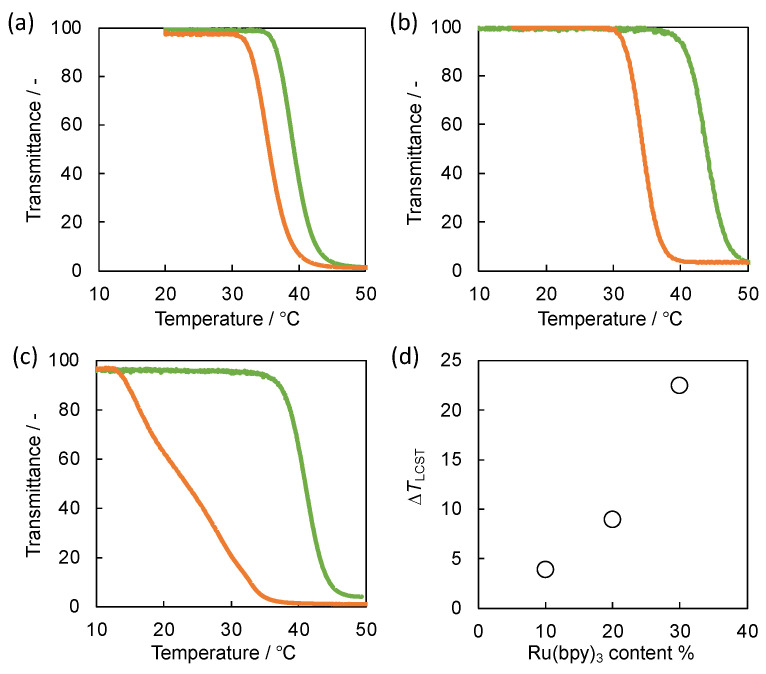
Temperature dependence of optical transmittance for the aqueous solutions containing (**a**) SOP-10, (**b**) SOP-20 and (**c**) SOP-30 in reduced state (orange) and oxidized state (green). (**d**) The dependence of ∆*T*_LCST_ on Ru(bpy)_3_ content.

Figure 4 displays the phase transition behavior of the self-oscillating polymers in both reduced and oxidized states in water. All the self-oscillating polymers exhibited a lower critical solution temperature (LCST) type behavior in both redox states, and the LCSTs of the reduced polymers were lower than those of the corresponding polymers in the oxidized state. The differences in LCSTs between the reduced and oxidized states (∆*T*_LCST_) originated from the increase of the hydrophilicity of the polymers by increasing the positive charge of the conjugated Ru(bpy)_3_ moiety [12]. ∆*T*_LCST_ was increased with increasing the amount of the immobilized Ru(bpy)_3_ moiety (Figure 4d). The result is consistent with our previous research [10]. The three self-oscillating polymers with different ∆*T*_LCST_ were successfully prepared.

### 2.3. Gelation Time Analysis

**Figure 5:**
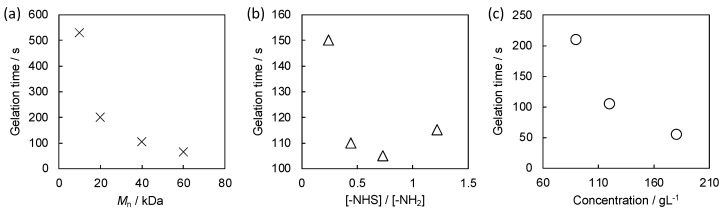
The dependence of the gelation time on (**a**) molecular weight of the polymeric crosslinkers, (**b**) [–NHS]/[–NH_2_] ratio and (**c**) concentration of pre-gel solution.

The gelation time of the self-oscillating gels fabricated by the polymer crosslinking method was investigated by modulating the molecular weight of the polymeric crosslinkers, the feeding ratio of the crosslinking moieties and the concentration of the pre-gel polymer solution. Figure 5a displays the dependence of the gelation time on the molecular weight of the polymeric crosslinkers. In this experiment, the self-oscillating linear polymer and the mixing ratio of the two polymer solutions were fixed to SOP-10 and 1:1 (*v*/*v*), respectively. The gelation time became shorter with the increase in molecular weight of the polymeric crosslinkers. The composition ratios of the synthesized polymeric crosslinkers were almost identical as shown in Table 2, thus the number of NHS moieties on the one polymeric crosslinker increases as the molecular weight increases. These results suggested that a large number of NHS moieties on the one polymeric crosslinker leads to the fast percolation of the polymer network at the early stage of the crosslinking process.

Then we evaluated the effect of the feeding ratio of the reactive groups, that is, amino groups on the self-oscillating polymers and NHS groups on the polymeric crosslinkers, on the gelation time. SOP-10 and PCL-40k were used for the experiment, and the concentration of each polymer solution was adjusted so that the concentration of the pre-gel mixture was 120 g L^−1^. As shown in Figure 5b, a local minimum appears on the gelation time with respect to the increase in the fed of the NHS groups. Because the local minimum was located around [-NHS]/[-NH_2_] = 1, the efficiency of crosslinking may decrease when the fed of either reactive group is excessive. For the fast gelation, the homogenous distribution of the complementary reactive sites in the pre-gel solution is of importance.

Figure 5c shows the dependence of the gelation time on the polymer concentration. We used SOP-10 and PCL-40k for this experiment, and the mixing ratio of the two polymer solutions was fixed to 1:1 (*v*/*v*). As the polymer concentration increased, the gelation time became shorter. In high concentrations, the interpenetration occurs between the polymers at the random coil state. The interpenetration between the self-oscillating linear polymers and the polymeric crosslinkers might facilitate the fast percolation of the polymer network. Finally, we revealed that the gelation time could be shortened to 50 s in the optimum condition.

### 2.4. Autonomous Swelling-Deswelling Behavior of Self-Oscillating Gels Fabricated by Polymer Crosslinking Method

#### 2.4.1. Effect of Polymer Composition on the Autonomous Swelling-Deswelling Behavior

The dynamic swelling-deswelling behavior of the self-oscillating gels fabricated by the polymer crosslinking method was investigated in the condition of BZ reaction. First, the cylindrical self-oscillating gels were fabricated using the self-oscillating linear polymers with various Ru(bpy)_3_ compositions and the polymeric crosslinker. In this experiment, the polymeric crosslinker and the mixing ratio of the two polymer solutions were fixed to PCL-40k and 1:1 (*v*/*v*), respectively. The self-oscillating gels composed of SOP-XX is abbreviated to SOG-XX.

**Figure 6:**
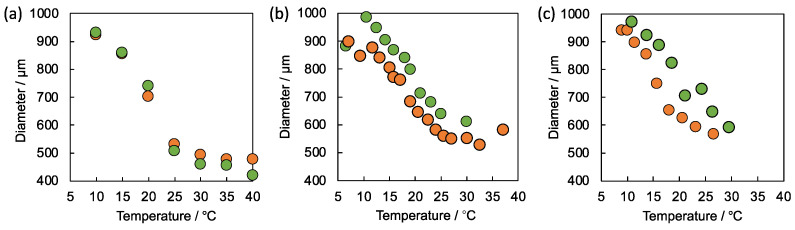
Equilibrium swelling behavior of (**a**) SOG-10, (**b**) SOG-20 and (**c**) SOG-30 in reduced state (orange) and oxidized state (green) at the various temperatures. The gels were equilibrated in water containing HNO_3_ (894 mM) and NaBrO_3_ (100 mM) for the oxidized state and in water containing HNO_3_ (894 mM) and NaCl (100 mM) for the reduced state.

Before evaluating the self-oscillating behavior of the gels, the swelling profiles of the equilibrated gels at various temperatures were measured in both the reduced and oxidized states (Figure 6a–c). In all cases, the self-oscillating gels show a volume phase transition with temperature changes in both redox states. Further, all the gels started shrinking from the lower temperature region compared to the LCSTs of the corresponding self-oscillating linear polymers. The results reflect the consumption of the hydrophilic amino groups on the self-oscillating linear polymers for crosslinking. The difference in the gel diameters between the two redox states became larger by increasing the composition of Ru(bpy)_3_ on the self-oscillating linear polymers. We previously reported that the difference in the gel diameters between the two redox states increases accompanying the increase in the amount of Ru(bpy)_3_ in the poly(NIPAAm-*co*-NAPMAm-*co*-NAPMAmRu) gels [10]. In this study, the composition of Ru(bpy)_3_ on the self-oscillating linear polymers corresponds to the net amount of Ru(bpy)_3_ contained in the self-oscillating gels. Thus, we concluded that the composition of Ru(bpy)_3_ on the self-oscillating linear polymers directly affects the equilibrium swelling behavior of the self-oscillating gels fabricated by the polymer crosslinking method.

**Figure 7:**
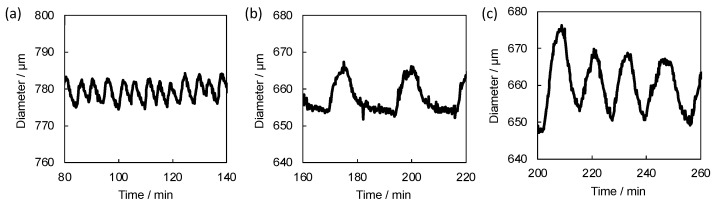
Time profiles of the hydrogel diameter of (**a**) SOG-10, (**b**) SOG-20 and (**c**) SOG-30 during BZ reaction at 20 °C. The catalyst-free BZ solution contains HNO_3_ (894 mM), NaBrO_3_ (100 mM) and malonic acid (64 mM) at 20 °C.

Then the dynamic swelling-deswelling behaviors of the gels were investigated in the BZ substrate solution (Figure 7a–c). All the self-oscillating gels exhibited autonomous volumetric oscillation in the catalyst-free BZ solution. The amplitude of the volumetric oscillation of the gels increased with increasing the composition of Ru(bpy)_3_ on the self-oscillating linear polymers. The result is in agreement with the equilibrium swelling behavior of the gels, i.e., the large difference in the gel diameters between the two redox states was observed for the gels composed of the self-oscillating linear polymers with high Ru(bpy)_3_ composition. It was suggested that the amplitude of the autonomous oscillation of the gels reflects the composition of Ru(bpy)_3_ on the self-oscillating linear polymers used for the gel formation. 

The oscillation period increased with changing the composition Ru(bpy)_3_ on the self-oscillating linear polymers from 10% to 20%, whereas the period decreased with changing the composition from 20% to 30%. In the conventional BZ reaction, the oscillation period increases with increasing the concentration of the catalyst [13]. We assumed that the difference in the oscillation period between SOG-20 and SOG-30 originates from the difference in the crosslinking densities. As the composition of Ru(bpy)_3_ on the self-oscillating linear polymers increases, the number of the unreacted amino groups decreases. Therefore, the crosslinking density becomes small when the self-oscillating linear polymers with high Ru(bpy)_3_ composition are used for the polymer crosslinking method. The lower crosslinking density of the self-oscillating gels may lead to the fast diffusion of the BZ substrate in the gels and shortened the oscillation period. Both the amount of Ru(bpy)_3_ and crosslinking density in the self-oscillating gels regulates the oscillation period.

#### 2.4.2. Effect of Polymer Mixing Ratio on the Autonomous Swelling-Deswelling Behavior

To evaluate the effect of the mixing ratio of the self-oscillating linear polymer and polymeric crosslinker, the self-oscillating gels were fabricated with various mixing ratios of the two solutions. The solutions of SOP-30 and PCL-40k were mixed in ratios of 1:1 (SOG-(1:1)), 1.5:1 (SOG-(1.5:1)) and 1:1.5 (SOG-(1:1.5)), and the mixtures were injected into glass capillaries to fabricate the corresponding gels. 

**Figure 8:**
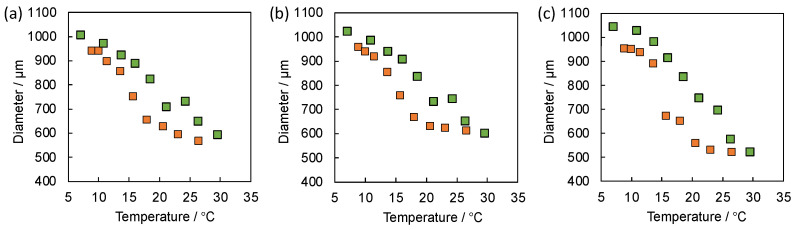
Equilibrium swelling behavior of (**a**) SOG-(1:1), (**b**) SOG-(1.5:1) and (**c**) SOG-(1:1.5) in reduced state (orange) and oxidized state (green) at the various temperatures. The gels were equilibrated in water containing HNO_3_ (894 mM) and NaBrO_3_ (100 mM) for the oxidized state and in water containing HNO_3_ (894 mM) and NaCl (100 mM) for the reduced state.

Figure 8a,b show the equilibrium swelling behavior of SOG-(1:1) and SOG-(1.5:1), respectively, in both the reduced and oxidized states at the various temperatures. Surprisingly, SOG-(1:1) exhibited almost the same swelling behavior as SOG-(1.5:1) in both two redox states, although the net amounts of Ru(bpy)_3_ in the gels were significantly different. These results suggested that the property of the self-oscillating linear polymer was dominant in the equilibrium swelling behavior of the self-oscillating gels fabricated in this condition. Sometimes, the polymer crosslinking method provides a hydrogel that strongly reflects a property of a linear polymer used for the gel formation due to the block-type sequence of the polymer network [14]. The phenomena are also consistent with the aforementioned results for the equilibrium swelling behavior of the self-oscillating gels fabricated by using the self-oscillating linear polymers with various Ru(bpy)_3_ contents. Compared to SOG-(1:1) and SOG-(1.5:1), SOG-(1:1.5) showed large differences in the gel diameters between the two redox states (Figure 8c). In the crosslinking condition, the NHS group was abundantly supplied for the crosslinking, thus it is expected that most of the amino groups on SOP-30 were consumed. We assumed that the consumption of the hydrophilic amino groups leads to a decrease in the LCSTs of the self-oscillating linear polymers and enhances the contraction of the gel, especially in the reduced state.

**Figure 9:**
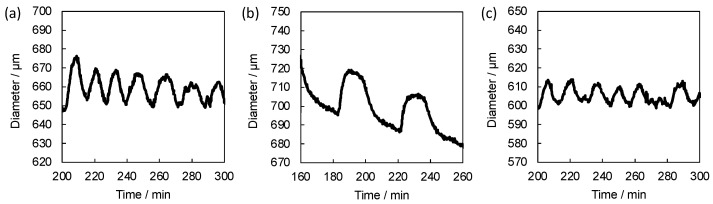
Time profiles of the hydrogel diameter of (**a**) SOG-(1:1), (**b**) SOG-(1.5:1) and (**c**) SOG-(1:1.5) during BZ reaction at 20 °C. The catalyst-free BZ solution contains HNO_3_ (894 mM), NaBrO_3_ (100 mM) and malonic acid (64 mM) at 20 °C.

Although the equilibrium swelling behavior of the self-oscillating gels strongly reflected the properties of the corresponding self-oscillating linear polymers, we revealed that the amplitudes of the dynamic volume oscillation of the gels were directly influenced by the net amount of Ru(bpy)_3_ in the whole gels (Figure 9a–c). The amplitudes increased as the ratio of the self-oscillating linear polymer to the polymeric crosslinker increased, that is, as the net amount of Ru(bpy)_3_ in the gels increased. This result is inconsistent with the expected result from the equilibrium swelling behavior of the gels. We concluded that the amplitude of the dynamic volume oscillation of the gels is simply regulated by the composition of the gels, independent of the properties of the constituent linear polymers and the equilibrium swelling behavior of the gels. This phenomenon implies that the factors dominating each behavior are different for equilibrium and dynamic behavior.

## 3. Conclusions

In this work, we successfully developed the polymer crosslinking method for the fabrication of the self-oscillating gels by utilizing a condensation reaction between amino groups and NHS esters. The gelation time became shorter as the molecular weight of the polymeric crosslinkers increased; the mixing ratio of the amino groups to the NHS groups gets closer to 1:1, and the concentration of the pre-gel solution increases. The equilibrium and self-oscillating behavior of the gels were investigated by modulating the compositions of self-oscillating linear polymers and the mixing ratio of the self-oscillating linear polymers to the polymeric crosslinkers. We revealed that the dynamic swelling-deswelling behavior of the gels was simply affected by the net amount of the Ru(bpy)_3_ in the whole gels, although the equilibrium swelling behavior was influenced by the properties of the constituent linear polymers. Our results bring the opportunity to access the origin of the dynamic and equilibrium behavior of materials by the hierarchical assembly as well as enable easy microfabrication of the self-oscillating gel.

## 4. Materials and Methods

### 4.1. Materials

*N*-*I*sopropylacrylaminde (NIPAAm) was kindly provided by KJ Chemicals (Tokyo, Japan) and purified by recrystallization from toluene/*n*-hexane. *N*-(3-Aminopropyl) methacrylamide hydrochloride (NAPMAm) was purchased from Polyscience (Warrington, PA, USA). Bis(2,2′-bipyridine)(1-(4′-methyl-2,2′-bipyridine-4-carbonyloxy)-2,5-pyrroli-dinedione)ruthenium(II) bis(hexafluorophosphate) (Ru-NHS) and *S*-1-dodecyl-*S*′-(α,α′-dimethyl-α″-acetic acid)-trithiocarbonate (DDMAT) were purchased from Trylead Chemical (Hangzhou, China). All other chemicals were purchased from Wako Pure Chemical Industries (Osaka, Japan). 

### 4.2. Preparation of the Self-Oscillating Linear Polymers

The self-oscillating linear polymers were synthesized according to our previous report [15]. A round-bottom flask was charged with DDMAT (182 mg, 0.5 mmol), NIPAAm (10.182 g, 90 mmol), NAPMAm (1.797 g, 10 mmol), V-65 (12.4 mg, 0.05 mmol)) and methanol (50 mL) and purged with Ar for 40 min. RAFT random copolymerization was then carried out at 60 °C for 4 h. The monomer conversions were determined by ^1^H-NMR measurement of the reaction mixture in D_2_O (Figure S1), recorded by an ECS400 spectrometer (JEOL Ltd., Tokyo, Japan). After the polymerization, the solvent was removed by evaporation. The reaction mixture was dissolved in acetone and was purified by reprecipitation from acetone/*n*-hexane as good/poor solvents. The reprecipitation process was repeated three times. The purified copolymer was dried under a vacuum at room temperature. The terminal trithiocarbonate groups of the observed copolymer were removed by the following procedure. The observed copolymer and AIBN (1 g) were dissolved in ethanol (190 mL), and the solution was purged with Ar for 30 min. The cleavage reaction was carried out at 70 °C for 16 h. The reaction mixture was concentrated by evaporation, purified by reprecipitation from acetone/*n*-hexane and then dried under vacuum at room temperature. 

Then, Ru-NHS was immobilized into the observed copolymer. The copolymer (1.018 g, 0.721 mmol as amino groups) was dissolved in dry DMSO (25 mL). A solution containing dry DMSO (25 mL), Ru-NHS and triethylamine (TEA) was mixed into the copolymer solution, and the mixed solution was stirred at room temperature for 24 h. The self-oscillating linear polymers with various loading amounts of Ru(bpy)_3_ were prepared by modulating the fed of Ru-NHS and TEA. The reaction mixtures were purified by dialysis against DMSO for 2 days and then water for 7 days. The observed polymer solution was freeze-dried to collect the self-oscillating linear polymer as solid. The Ru(bpy)_3_ content in the copolymer was determined by UV-vis spectroscopy (Figure S2) (UV-2500 PC, Shimadzu, Kyoto, Japan). The amount of immobilized Ru(bpy)_3_ was calculated from the absorbance at 465 nm using a calibration curve which we reported [10]. The chemical structure of the self-oscillating linear polymer was confirmed by attenuated total reflection Fourier transform infrared (ATR/FT-IR) measurement (Figure S3) by IRSprit (Shimadzu, Co., Kyoto, Japan) with a diamond ATR accessory (GladiATR^TM^, PIKE technologies, Inc., WI, USA). The peaks at 1535 cm^−1^ and 1637 cm^−1^ corresponded to the absorptions of the amide N-H and C=O of NIPAAm, respectively. The peaks that appeared around 2971 cm^−1^ and 3269 cm^−1^ also corresponded to the characteristic absorptions of NIPAAm, C-H and N-H stretching, respectively. The peak at 843 cm^−1^ corresponded to the absorptions of the C-H deformation of bipyridine in Ru(bpy)_3_. The polydispersity index (PDI) of the self-oscillating linear polymers was determined by the HLC-8220 gel permeation chromatography (GPC) system (Figure S4) (Tosoh corporation, Tokyo, Japan), using DMF containing LiCl (50 mM) as a mobile phase. The GPC columns were calibrated using poly(ethylene glycol) standards.

### 4.3. Preparation of the Polymer Crosslinkers

The polymeric crosslinkers were synthesized according to our previous report [10]. The polymer crosslinkers with various molecular weights were prepared by modulating the polymerization time and the feeding ratio of the monomers and DDMAT, a chain transfer agent for RAFT polymerization. Briefly, a round-bottom flask was charged with DDMAT, *N*-acryloxysuccinimide (NAS), NAPMAm, AIBN and 1,4-dioxane and purged with Ar for 30 min. RAFT random copolymerization was then carried out at 65 °C. The monomer conversions were determined by ^1^H-NMR measurement of the reaction mixture in CDCl_3_ (Figures S5–S8), recorded by an ECS400 spectrometer (JEOL Ltd., Tokyo, Japan). After the polymerization, the solvent was removed by evaporation. The reaction mixture was dissolved in acetone and was purified by reprecipitation from acetone/*n*-hexane as good/poor solvents. The reprecipitation process was repeated three times. The purified copolymer was dried under a vacuum at room temperature. The terminal trithiocarbonate groups of the observed copolymer were removed by the following procedure. The observed copolymer and AIBN (1 g) were dissolved in 1,4-dioxane (50 mL), and the solution was purged with Ar for 30 min. The cleavage reaction was carried out at 70 °C for 16 h. The reaction mixture was concentrated by evaporation, purified by reprecipitation from acetone/*n*-hexane and then dried under vacuum at room temperature. The chemical structure of the polymeric crosslinker was confirmed by attenuated total reflection Fourier transform infrared (ATR/FT-IR) measurement (Figure S9) by IRSprit (Shimadzu, Co., Kyoto, Japan) with a diamond ATR accessory (GladiATR^TM^, PIKE technologies, Inc., WI, USA). The peaks at 1533 cm^−1^ and 1641 cm^−1^ corresponded to the absorptions of the amide N-H and C=O of NIPAAm, respectively. The peaks that appeared around 2971 cm^−1^ and 3300 cm^−1^ also corresponded to the characteristic absorptions of NIPAAm, C-H and N-H stretching, respectively. The peak at 1736 cm^−1^ corresponded to the absorptions of the C=O of succinimide moiety. PDI of the polymer crosslinkers was determined by the HLC-8220 GPC system (Figure S10) (Tosoh corporation, Tokyo, Japan), using DMF containing 50 mM LiCl as a mobile phase. The GPC columns were calibrated using poly(ethylene glycol) standards.

### 4.4. LCST Analysis of the Self-Oscillating Polymers

The optical transmittance of the self-oscillating polymer solution at various temperatures in each redox state was recorded by using a UV-vis spectrophotometer (UV-2500 PC, Shimazu, Japan, monitored at 583.5 nm). The self-oscillating polymers were dissolved in water containing HNO_3_ (894 mM) and NaBrO_3_ (100 mM) for the measurement of the oxidized state and in water containing HNO_3_ (894 mM) and NaCl (100 mM) for the measurement of reduced state. NaCl was added to maintain the ionic strength of the solution. The onset of transmittance change was used in estimating LCST. LCST of the self-oscillating polymer was defined as the temperature when the transmittance of the solution reaches 90%.

### 4.5. Gelation Time Analysis

The gelation time by the polymer crosslinking method was evaluated by pipetting method [16]. Each self-oscillating linear polymer and polymer crosslinker was separately dissolved in dry DMSO; 30 µL of the two solutions were injected into a 2.2 mL screw vial, and the pre-gel mixture was pipetted up and down with 200 µL tips until the tip became clogged. We defined the time to reach the clogged state as “gelation time” in this work.

### 4.6. Fabrication of Cylindrical Self-Oscillating Gels by Polymer Crosslinking Method

Each self-oscillating linear polymer and polymer crosslinker was separately dissolved in dry DMSO. The two solutions were mixed, and the mixture was injected into a glass capillary with an inner diameter of 1.02 mm. The concentration of each polymer solution was adjusted so that the concentration of the pre-gel mixture was 120 g L^−1^. The mixing ratio of the polymer solutions was adjusted for each experiment. After the gelation via the post-polymerization crosslinking, the prepared gels were dialyzed against DMSO and were gradually replaced with water in the glass capillary. The gels were moved to a hot water bath (70 °C) to remove the glass capillary by shrunken the hydrogel. The prepared gels were dialyzed against water further for 7 days to remove unreacted components.

### 4.7. Volume Phase Transition Temperature Analysis of the Self-Oscillating Gels 

The cylindrical self-oscillating gels were equilibrated in water containing HNO_3_ (894 mM) and NaBrO_3_ (100 mM) for the measurement of the oxidized state and in water containing HNO_3_ (894 mM) and NaCl (100 mM) for the measurement of reduced state. NaCl was added to maintain the ionic strength of the solution. The photos of the cylindrical self-oscillating gels at the various temperature points in each redox state were recorded by an optical microscope (VHX 900, Keyence, Osaka, Japan). The acquired images were analyzed with ImageJ software (NIH) to calculate the gel diameters. 

### 4.8. Observation of the Autonomous Swelling-Deswelling Behavior of the Self-Oscillating Gels 

The cylindrical self-oscillating gels were immersed in a catalyst-free BZ solution containing HNO_3_ (894 mM), NaBrO_3_ (100 mM) and malonic acid (64 mM) at 20 °C. The time profiles of the gel diameter during self-oscillation were observed from images recorded by an optical microscope (VHX 900, Keyence, Osaka, Japan ). The images were analyzed by ImageJ software.

## Figures and Tables

**Table 1:** Summary of the self-oscillating linear polymers.

Sample	Polymer Formula	*M*_n_/kDa ^1^	PDI ^2^
SOP-10	P(NIPAAm_0.90_-*co*-NAPMAm_0.09_-*co*-NAPMAmRu_0.01_)_174_	21	1.90
SOP-20	P(NIPAAm_0.90_-*co*-NAPMAm_0.08_-*co*-NAPMAmRu_0.02_)_174_	22	1.43
SOP-30	P(NIPAAm_0.90_-*co*-NAPMAm_0.07_-*co*-NAPMAmRu_0.03_)_174_	23	1.42

^1^ Determined by ^1^H-NMR measurement. ^2^ Determined from GPC traces.

**Table 2:** Summary of the polymeric crosslinkers.

Sample	Polymer Formula	*M*_n_/kDa ^1^	PDI ^2^
PCL-10k	P(NIPAAm_0.93_-*co*-NAS_0.07_)_89_	10	1.33
PCL-20k	P(NIPAAm_0.93_-*co*-NAS_0.07_)_175_	20	1.31
PCL-40k	P(NIPAAm_0.93_-*co*-NAS_0.07_)_333_	39	1.24
PCL-65k	P(NIPAAm_0.93_-*co*-NAS_0.07_)_554_	65	1.26

^1^ Determined by ^1^H-NMR measurement. ^2^ Determined from GPC traces.

## Data Availability

Not applicable.

## Supplementary Materials

The following are available online at: https://www.mdpi.com/article/10.3390/gels8050267/s1, Figure S1: 1H-NMR spectrum of the reaction mixture for the synthesis of P(NIPAAm-co-NAPMAm) in D2O. Figure S2: UV-Vis absorption spectra of SOP-10, SOP-20 and SOP-30 in water. Figure S3: FT-IR spectrum of SOP-30. Figure S4: GPC traces of the self-oscillating linear polymers. Figures S5–S8: 1H-NMR spectra of the reaction mixture for the synthesis of polymeric crosslinkers in CDCl3. Figure S9: FT-IR spectrum of PCL-10k. Figure S10: GPC traces of the polymeric crosslinkers.
