# Supplementary material for: Fabrication of Self-Oscillating Gels by Polymer Crosslinking Method and Analysis on Their Autonomous Swelling-Deswelling Behaviors
Source: Gels. 2022 Apr 24;8(5):267. doi: 10.3390/gels8050267 (PMC9141476; doi:10.3390/gels8050267)
Supplement: Supplementary file 1 [file gels-08-00267-s001.zip › gels-1667555-supplementary.pdf]

gels

Supplementally Materials

Fabrication of Self-oscillating Gels by Polymer Crosslinking Method and Analysis on Their Autonomous Swelling -deswelling Behavior

Komi Sato†, Takafumi Enomoto†, Aya M. Akimoto and Ryo Yoshida*

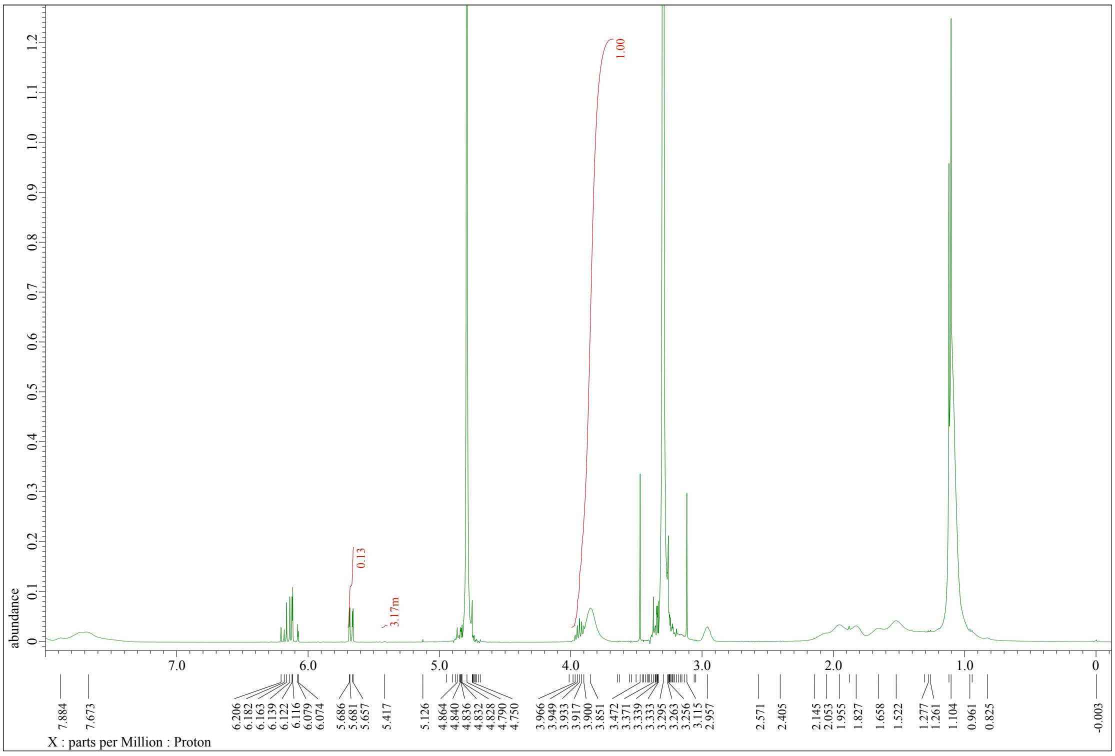

Figure S1. ^{1}H-NMR spectrum of the reaction mixture for the synthesis of P(NIPAAm-*co*-NAPMAm) in D_2O.

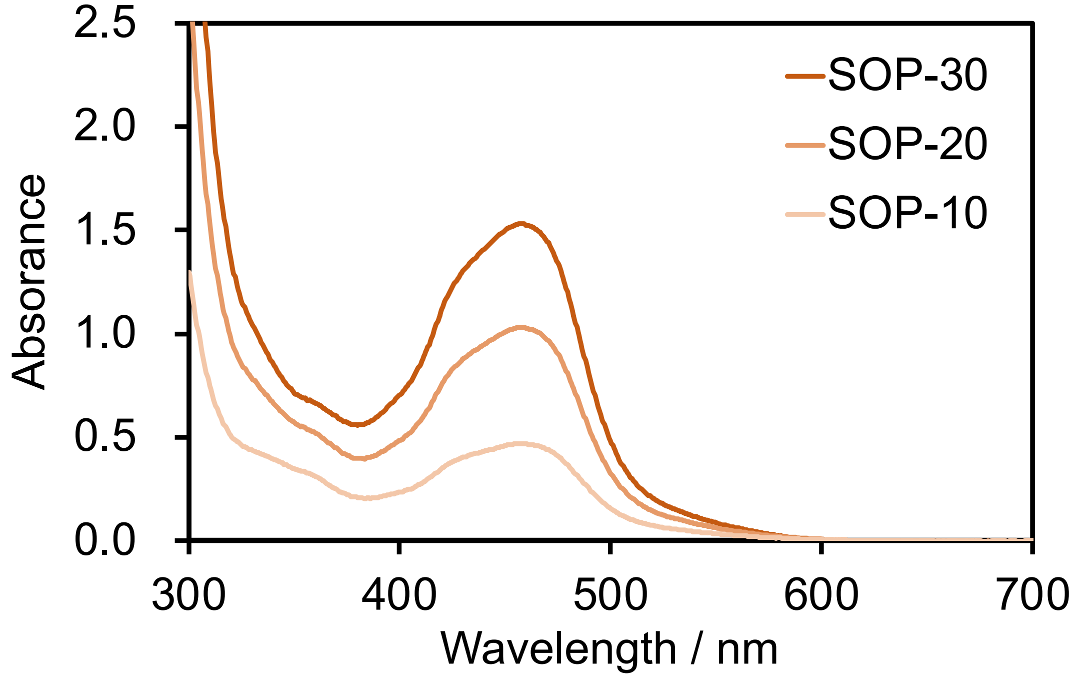

Figure S2. UV-Vis absorption spectra of SOP-10, SOP-20 and SOP-30 in water.

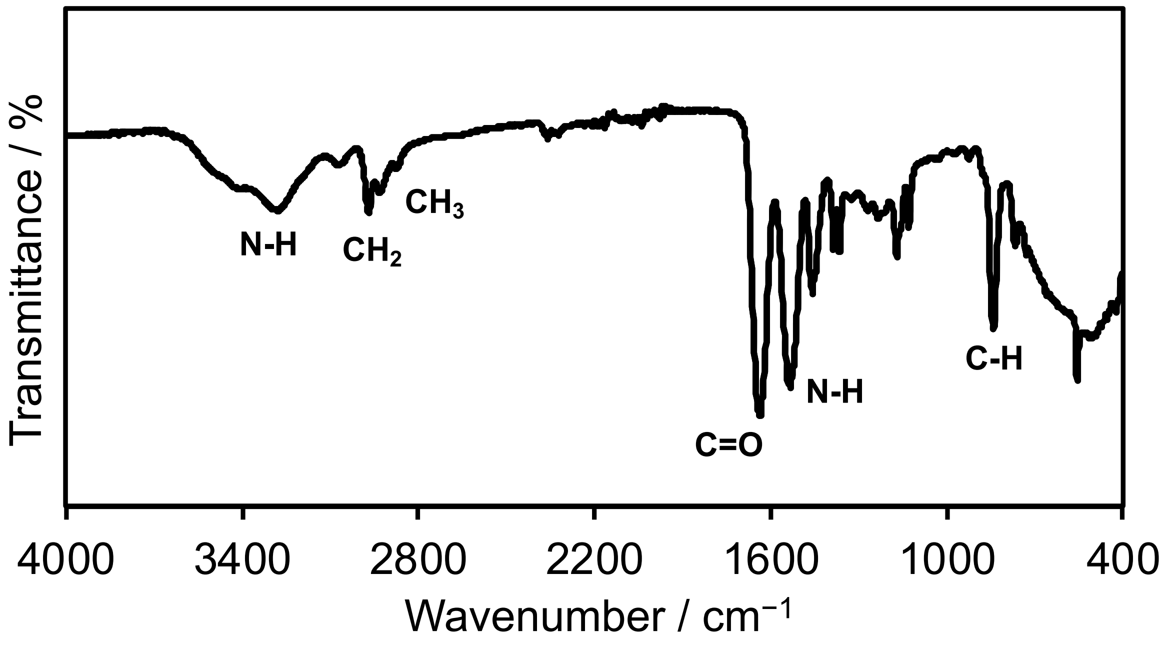

Figure S3. FT-IR spectrum of SOP-30.

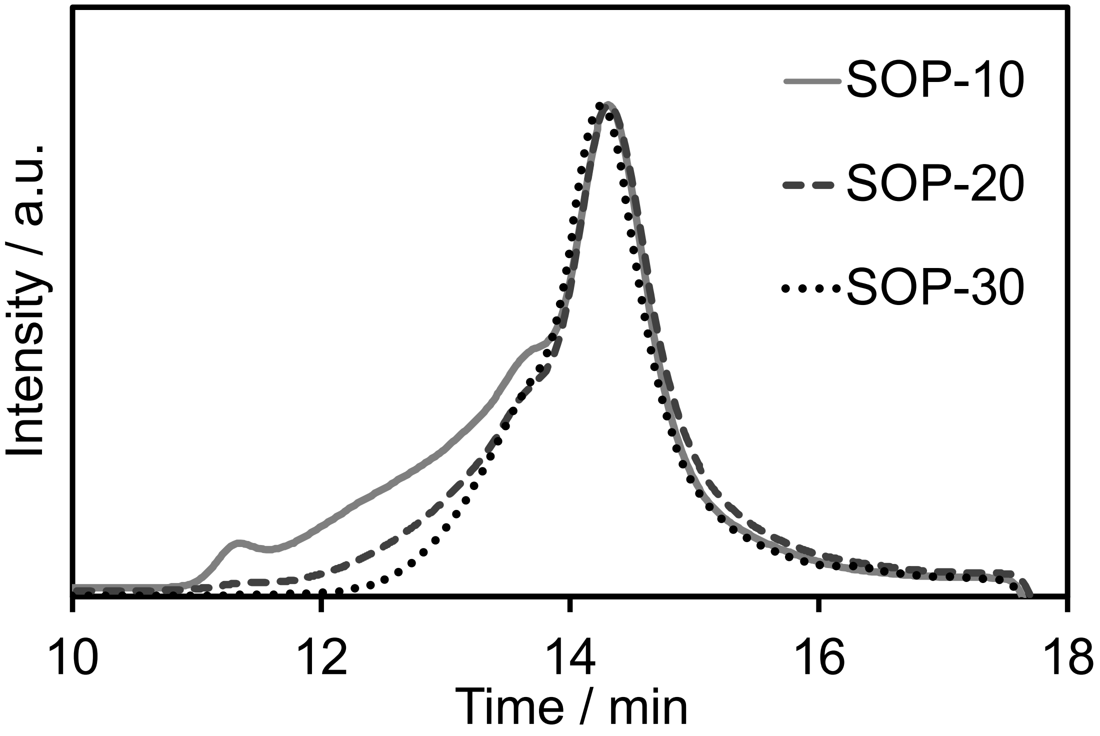

Figure S4. GPC traces of the self-oscillating linear polymers.

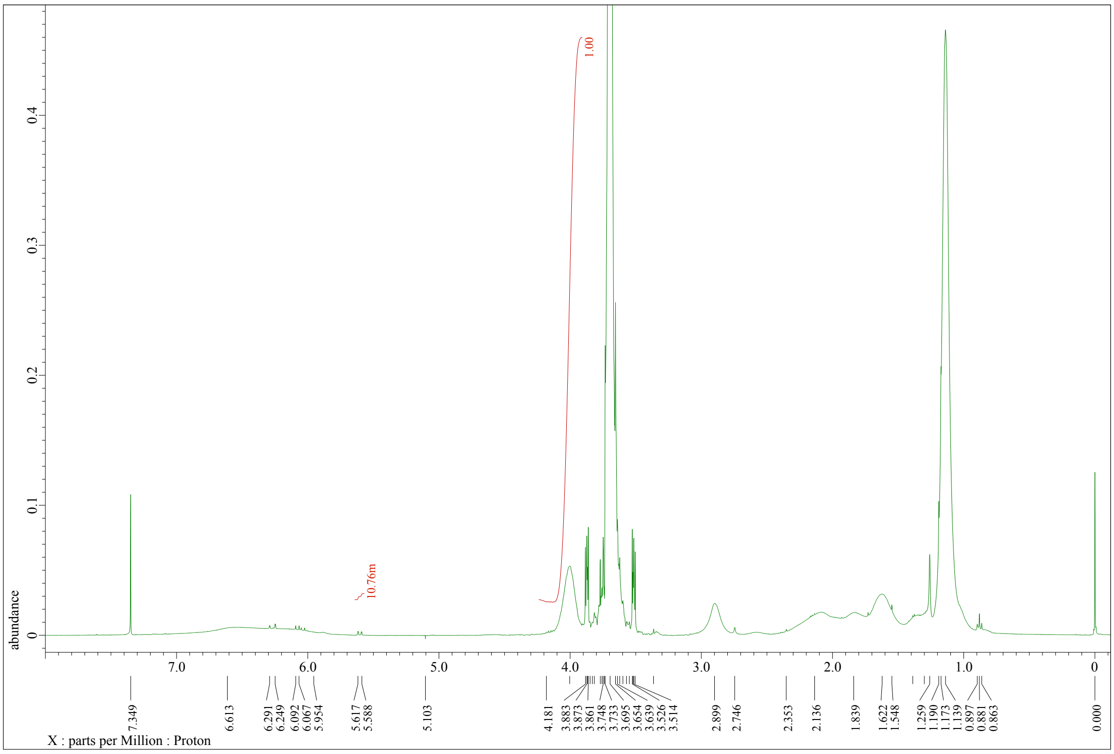

Figure S5. ^{1}H-NMR spectrum of the reaction mixture for the synthesis of PCL-10k in $CDCl_3$.

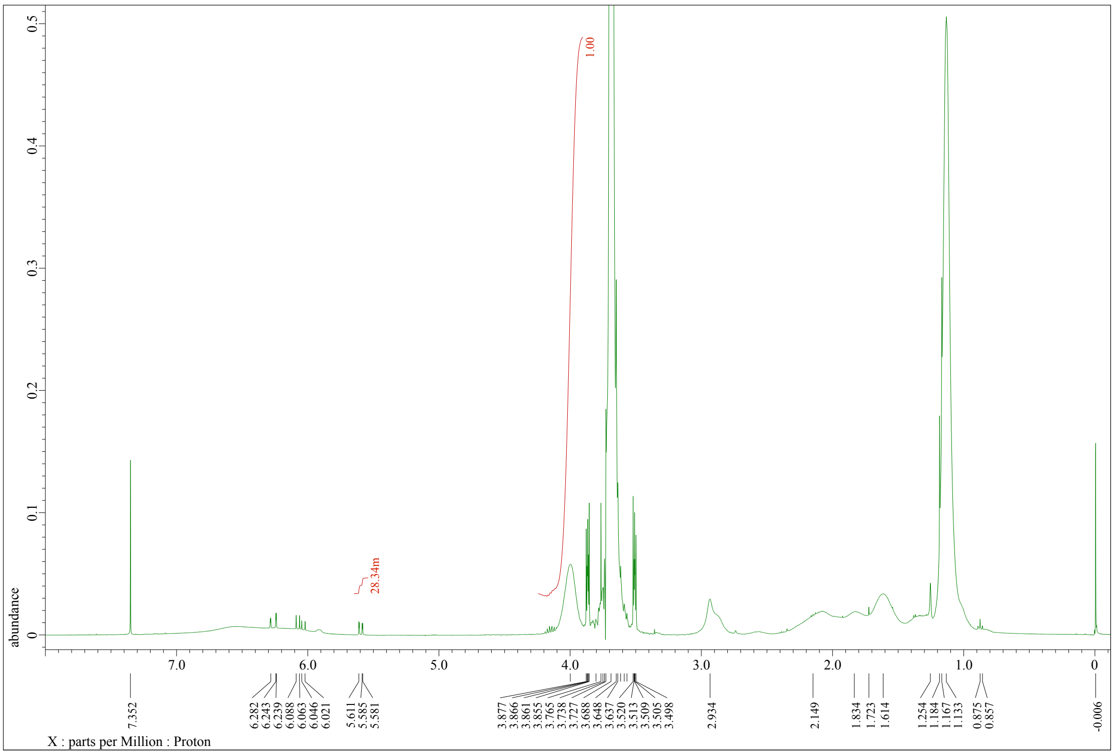

Figure S6. ¹H-NMR spectrum of the reaction mixture for the synthesis of PCL-20k in $CDCl_3$.

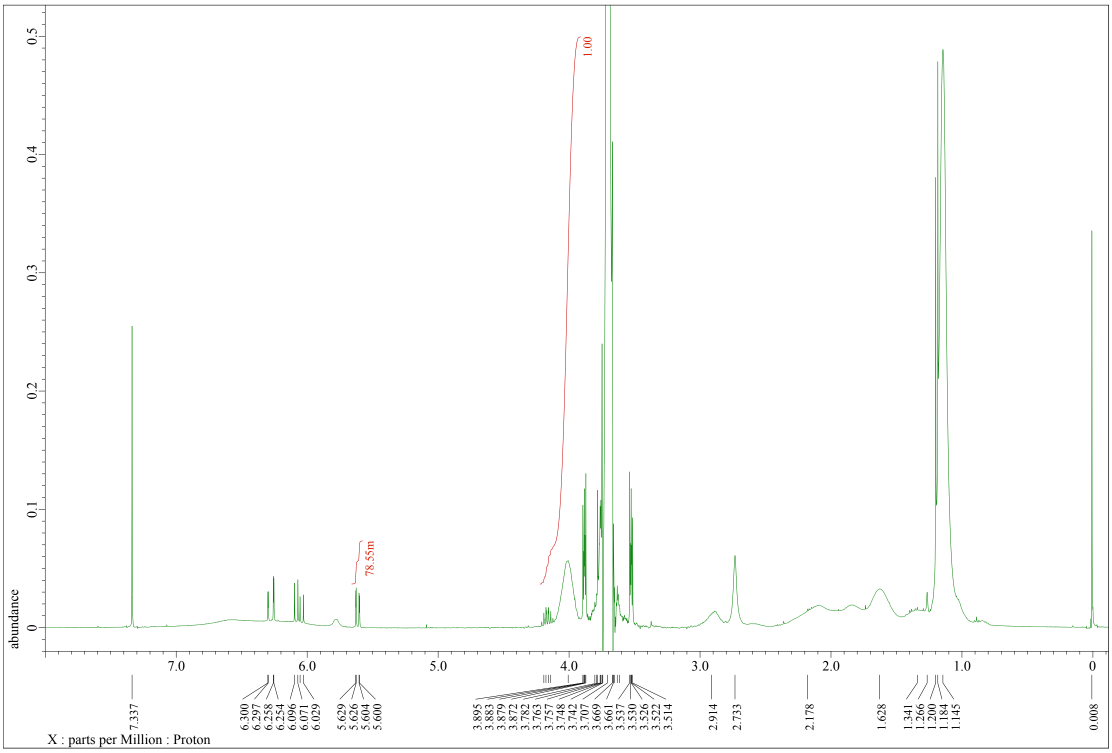

Figure S7. ¹H-NMR spectrum of the reaction mixture for the synthesis of PCL-40k in $CDCl_3$.

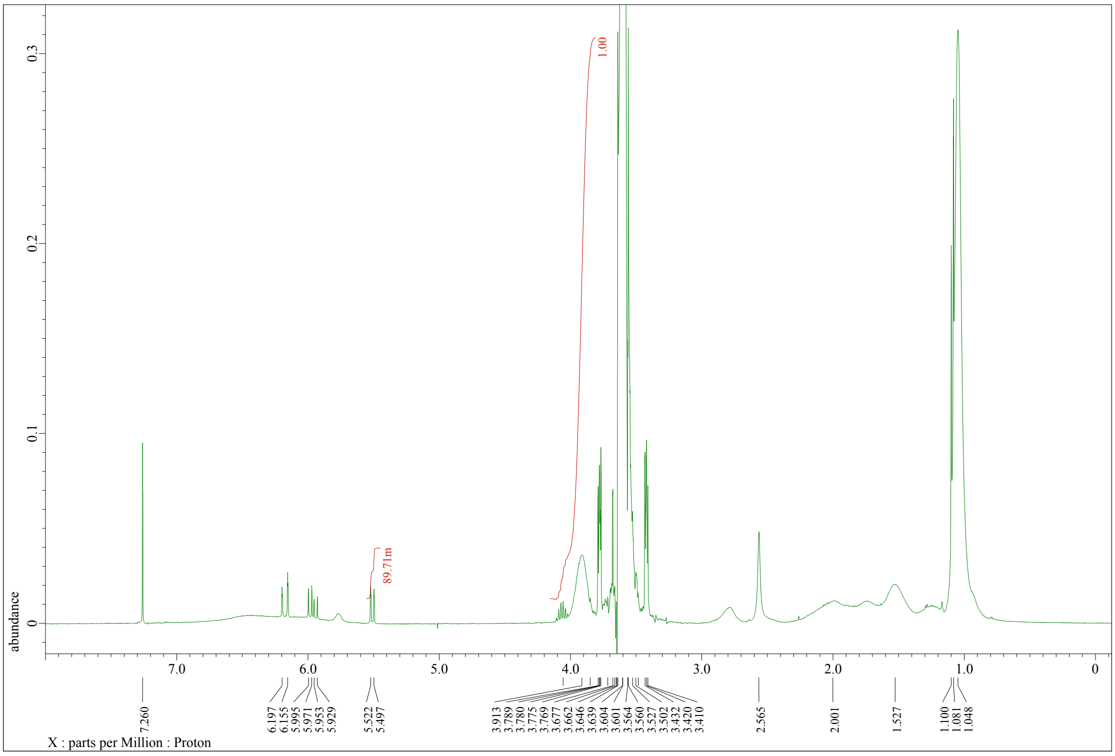

Figure S8. ^{1}H-NMR spectrum of the reaction mixture for the synthesis of PCL-65k in $CDCl_3$.

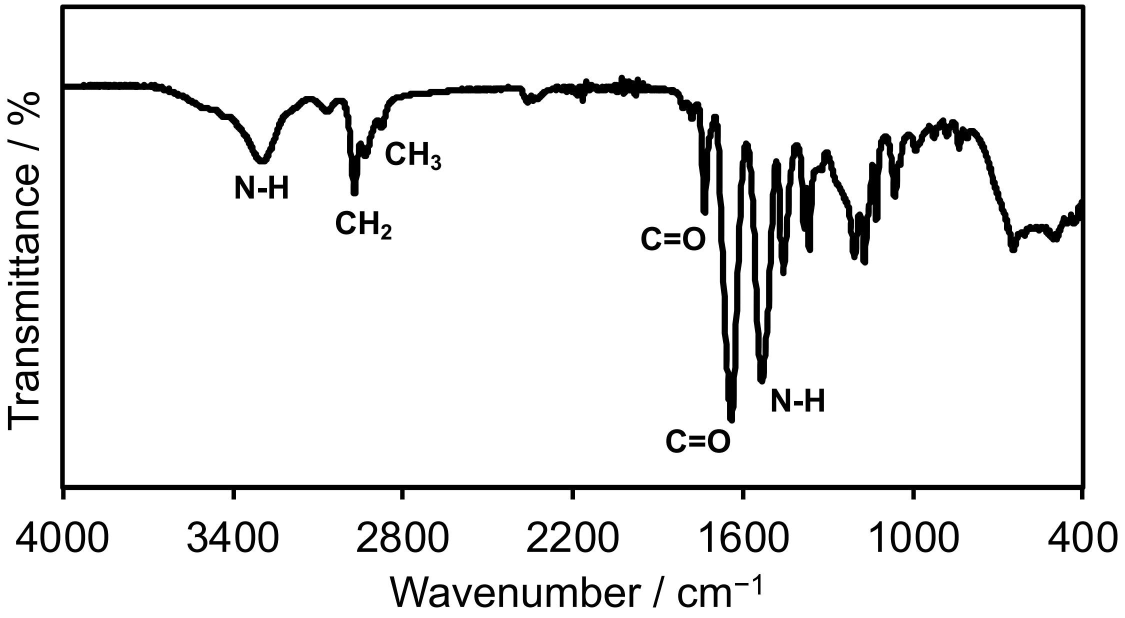

Figure S9. FT-IR spectrum of PCL-10k.

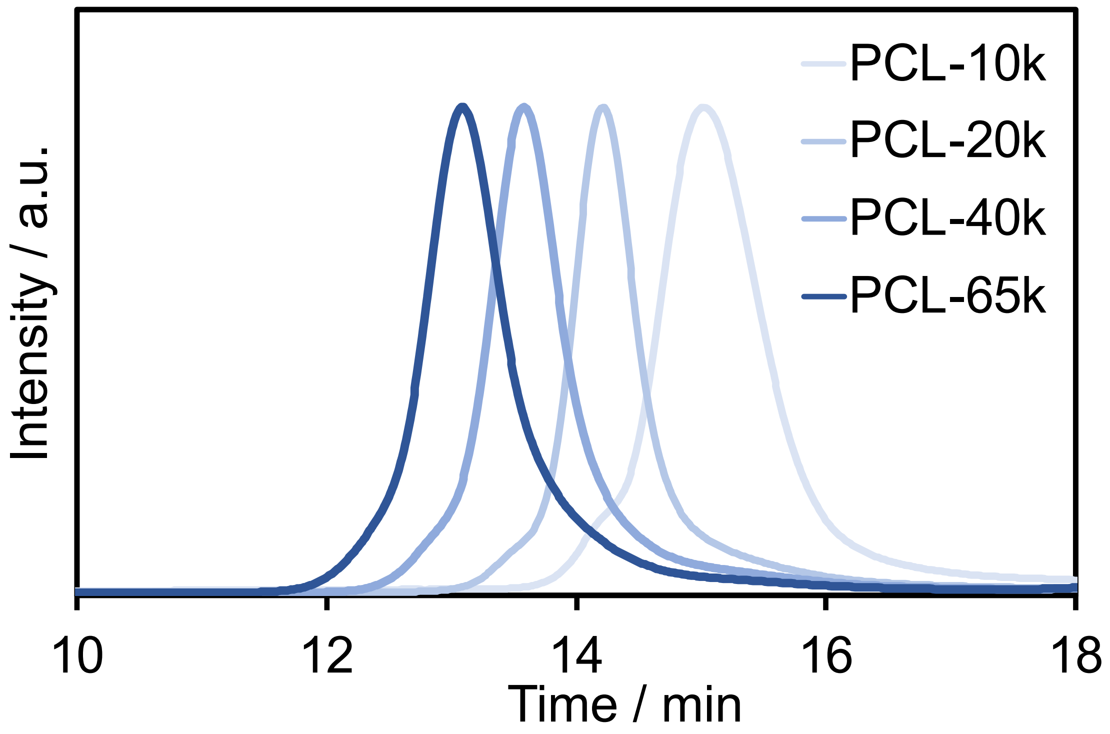

Figure S10. GPC traces of the polymeric crosslinkers.
